# Weighted *K*-means support vector machine for cancer prediction

**DOI:** 10.1186/s40064-016-2677-4

**Published:** 2016-07-25

**Authors:** SungHwan Kim

**Affiliations:** Department of Statistics, Korea University, Anam-dong, Seoul, 136-701 South Korea

**Keywords:** Support vector machine, *K*-means clustering, Weighted SVM, TCGA

## Abstract

To date, the support vector machine (SVM) has been widely applied to diverse bio-medical fields to address disease subtype identification and pathogenicity of genetic variants. In this paper, I propose the weighted *K*-means support vector machine (wKM-SVM) and weighted support vector machine (wSVM), for which I allow the SVM to impose weights to the loss term. Besides, I demonstrate the numerical relations between the objective function of the SVM and weights. Motivated by general ensemble techniques, which are known to improve accuracy, I directly adopt the boosting algorithm to the newly proposed weighted KM-SVM (and wSVM). For predictive performance, a range of simulation studies demonstrate that the weighted KM-SVM (and wSVM) with boosting outperforms the standard KM-SVM (and SVM) including but not limited to many popular classification rules. I applied the proposed methods to simulated data and two large-scale real applications in the TCGA pan-cancer methylation data of breast and kidney cancer. In conclusion, the weighted KM-SVM (and wSVM) increases accuracy of the classification model, and will facilitate disease diagnosis and clinical treatment decisions to benefit patients. A software package (wSVM) is publicly available at the R-project webpage (https://www.r-project.org).

**Electronic supplementary material:**

The online version of this article (doi:10.1186/s40064-016-2677-4) contains supplementary material, which is available to authorized users.

## Introduction

Cutting-edge microarray and sequencing techniques for transcriptome and DNA methylome have received increasing attentions to decipher biological processes and to predict the multi-causes of complex diseases [e.g., cancer diagnosis (Ramaswamy et al. 2001), prognosis (Vijver et al. 2002), and therapeutic outcomes (Ma et al. 2004)]. To this end, the supervised machine learning has considerably contributed to developing tools towards the translational and clinical application. For example, diverse biomarker panels on the basis of transcriptional expressions have been released [e.g. MammaPrint (van ’t Veer 2002), Oncotype DX (Paik et al. 2004), Breast Cancer Index BCI (Zhang et al. 2013) and PAM50 (Parker et al. 2009)] for survival, recurrence, drug response and disease subtypes. It is evident that effective prediction tasks advance clinical diagnosis tools that build on translating models from transcriptomic studies. In this standpoint, rapid and precise classification rules are imperative to support exploring disease-related biomarkers, diagnosis and sub-types identification, and to deliver meaningful information for tailored treatment and precision medicine.

The support vector machine (SVM) was originally introduced by Cortes and Vapnik (1995). Over the decades, the SVM has been applied to a range of study fields, including pattern recognition (Kikuchia and Abeb 2005), disease subtype identification (Gould et al. 2014), pathogenicity of genetic variants (Kircher et al. 2014) and so on. In theory, the forte of the SVM is attributed to its flexibility and outstanding classification accuracy. However, the SVM relies on the quadratic programming (QP), whose computational complexity is commonly costly and subject to size of data. Some methods to circumvent this drawback (Wang and Wu 2005; Lee et al. 2007) were proposed to speed up its computation with minimizing loss of accuracy. Interestingly, Wang and Wu (2005) applied the SVM to centers of *K*-means clustering alone (KM-SVM). Due to small cluster size *K*, this method dramatically diminishes the number of observations, and hence can reduces the high-computational cost. The KM-SVM assumes that cluster centers adequately account for original data. This KM-SVM is also called the Global KM-SVM (Lee et al. 2007) in short. Similarly, Lee et al. (2007) also proposed so-called the By-class KM-SVM, where class labels separate samples into two groups at the outset, to which I apply *K*-means clustering respectively, while the Global KM-SVM, in contrast, employs a majority voting to determine class labels of respective centers. Not surprisingly, it is commonplace that the KM-SVM performs worsen than the standard SVM in most cases. In other words, the KM-SVM pursues computational efficiency at the expense of prediction accuracy.

Yang et al. (2007) and Bang and Jhun (2014) proposed the weighted support vector machine and the weighted KM-SVM to improve accuracy in the context of the outlier sensitivity problem (i.e., WSVM-outlier). The primary idea is to assign weights to each data sample, which manipulates relative importance. It is proved that WSVM-outlier reduces the effect of outliers, and yields higher classification rates. Yet I notice that the WSVM-outlier solely adopts outlier-sensitive algorithms (e.g., a robust fuzzy clustering, kernel-based possibilistic c-means), that are only well-suited to adjusting outlier effects, but not always guarantees to perform best in general cases. It is, therefore, interesting to add other weight schemes applicable to general scenarios.

Boosting is a machine learning ensemble algorithm, making it possible to reduce bias and variance, and to boost predictive power. More specifically, most boosting algorithms (Schapire 1990; Breiman 1998; Freund and Schapire 1997) iteratively glean weak classifiers, and incorporate them to a strong classifier. At each iteration, weak classifiers gain weights in some reasonable ways, and thereby subsequent weak learners focus more on samples that preceding weak learners mis-classified. Over the decades, many have introduced diverse boosting algorithms: Schapire (1990) originally proposed (a recursive majority gate formulation), and Mason et al. (2000) developed boost by majority. Interestingly, Freund and Schapire (1997) then developed AdaBoost.M1, an adaptive algorithm known to be superior to the previous ones.

Taking all things into consideration, I proposed a new algorithm, the weighted KM-SVM (wKM-SVM) and weighted support vector machine (wSVM) to improve the KM-SVM (and SVM) via weights, together with the boosting algorithm. In this paper, I utilize AdaBoost.M1 (Freund and Schapire 1997) in place of the outlier-sensitive algorithms used in WSVM-outlier (Yang et al. 2007). The wKM-SVM (wSVM) adds weights to the hinge loss term, making it straightforward to derive the quadratic programming (QP) objective function, while the WSVM-outlier, to the contrary, directly maneuvers the penalization constant corresponding to each sample. Yang et al. (2007) hardly enables to grasp how each weight is implemented in optimization, whereas my proposed wKM-SVM (wSVM) can demonstrate the numerical relationship between the objective function and weights. The weighted KM-SVM (wKM-SVM) is universally applicable to many different data analysis scenarios, for which comprehensive experiments assess accuracy and provide comparisons with other methods.

In this paper, I applied the proposed method to pan-cancer methylation data (https://tcga-data.nci.nih.gov/tcga/) including breast cancer (breast invasive carcinoma) and kidney cancer (kidney renal clear cell carcinoma). From simulations and real applications, the proposed wKM-SVM (wSVM) is shown to be more efficient in predictive power, as compared to the standard SVM and KM-SVM, including but not limited to many popular classification rules (e.g., decision trees and k-NN and so on). In conclusion, the wKM-SVM (and wSVM) increases accuracy of the classification model that will ultimately improve disease understanding and clinical treatment decisions to benefit patients.

This paper is outlined as follows. In “Backgrounds” section, I review background studies in terms of the SVM and ensemble methods. In “Proposed methods” section, the weighted SVM algorithm is proposed. In “Numerical studies” section, I compare performance of my proposed methods with other methods, and claim biological implications from analysis of the TCGA pan-cancer data. In “Conclusion and discussion” section, conclusions and further studies are discussed.

## Backgrounds

### Support vector machine

Consider the data of $(x_1, y_1),\ldots , (x_N, y_N)$, with $x_n \in \chi \subset {\mathbb {R}}^m$ and $y_n\in \{-1,1\}$ for $n=1,\ldots, N$, where $\chi$ denotes an input space. Let $x_n$ and $y_n$ be an input and class label of the *n*th sample. $\langle \cdot ,\cdot \rangle$ and $\Vert \cdot \Vert$ denote the inner product and norm in ${\mathbb {R}}^m$. Define hyperplane by $f(x_n)=\langle w,x_n\rangle +b$. A classification rule that builds on *f*(*x*) is

$$\begin{aligned} G(x)=\text {sign}[f(x)]. \end{aligned}$$

Commonly, *w* and *b* are called the weight vector and bias. The optimal vector and bias can be obtained by solving the following quadratic optimization problem,

1 $$\begin{aligned} min_{w,b} \frac{1}{2} \Vert w \Vert ^2 + C \sum _{n=1}^N \xi _n, \end{aligned}$$

subject to $y_n(\langle w, x \rangle +b ) \ge 1- \xi _n$, $\xi \ge 0$ for $n=1,\ldots,N$, where $\xi _n$ are slack variables and *C* is the regularization parameter. Note that (1) can be reformulated with the Wolfe dual form by introducing the Lagrange multipliers.

2 $$\begin{aligned}&\text {argmax}_\alpha \frac{1}{2} \sum _{n=1}^N \sum _{m=1}^N y_n y_m \alpha _n \alpha _m \langle x_n, x_m \rangle - \sum _{n=1}^N \alpha _n, \nonumber \\&\text {subject to} \ \sum _{n=1}^N y_n \alpha _n = 0 \quad \text {and} \quad 0 \le \alpha _n \le C, \end{aligned}$$

where $\alpha _n$ is the Lagrange multiplier with respect to $x_n$ for $n=1,\dots ,N$. ${\hat{\alpha }}_n$ is then the solution of (2). From the derivatives of the Lagrange equations, I see that the solution of *f*(*x*) as below:

$$\begin{aligned} {\hat{f}}(x) = \sum _{n=1}^N {\hat{\alpha }}_n y_n \langle w , x \rangle + {\hat{b}}. \end{aligned}$$

Importantly, ${\hat{\alpha }}_n$ ($1 \le n \le N$) is a non-zero solution and its properties are induced by the Karush–Kuhn–Tucker conditions including boundary constraints. Taken together, the decision rule can be formed as

$$\begin{aligned} G(x)&=sign[f(x)] \\&= sign[\langle {\hat{w}} , x \rangle + {\hat{b}}]. \end{aligned}$$

For nonlinear decision rules, a kernel method can be applicable with the inner product $\langle \cdot ,\cdot \rangle$ replaced by a nonlinear kernel, $k(\cdot ,\cdot )$. For more details, see Cortes and Vapnik (1995).

### *K*-means SVM

The support vector machine using the *K*-means clustering (KM-SVM) is the SVM algorithm sequentially combined with the *K*-means clustering. Importantly, it is believed that the *K*-means clustering is one of the most popular clustering methods. The following describes how to implement KM-SVM. I first divide samples of train data into several clusters by applying the *K*-means clustering. Given pre-defined *K*, the *K*-means clustering produces clusters $C_1,\ldots , C_K$. Class labels (i.e., $-1$ or 1) of $C_n$ are assigned via majority voting ($1 \le n \le K$). Second, I build up a SVM classifier over derived cluster centers. It is interesting to note that the KM-SVM greatly cut down the number of data and support vectors used to estimate solutions, and so has the forte of computational efficiency. Wang and Wu (2005) originally introduced the prototype KM-SVM (Global KM-SVM). Due to its practical utilities, diverse KM-SVM-type classification rules have been proposed afterward (Gu and Han 2013; Lee et al. 2007). In this paper, I mainly focus on the KM-SVM methods proposed by Lee et al. (2007). Wang and Wu (2005) applies the *K*-means clustering to whole input data, while Lee et al. (2007) uses the *K*-means clustering to two sample groups independently separated by each class label (By-class KM-SVM). It is known that the By-class KM-SVM improves error rates, and efficiently circumvents the problem of imbalanced class labels.

## Proposed methods

### Weighted support vector machine

In this section, I newly introduce the weighted SVM that can accommodate some weights. The previous weighted SVMs (Yang et al. 2007; Bang and Jhun 2014) directly maneuver the penalization constant corresponding to each sample. With these strategies, I hardly grasp how each weight plays a role in optimization, leading to challenges to verify the numerical relationship between the objective function and weights. To the contrary, my proposed method adds weights to the hinge loss term, making it tractable to derive the quadratic programming (QP) objective function, and to impose weights to the hinge loss. In short, I call this the weighted KM-SVM (wKM-SVM) henceforth. In what follows, I formulate the SVM objective function with the penalization form:

3 $$\begin{aligned}&f(x) = \langle w, x \rangle +b, \nonumber \\&\text {min}_{w,b}\sum _{n=1}^N c_n [1- (y_n \langle w , x_n \rangle + b)]_+ + \lambda \Vert w \Vert ^2, \end{aligned}$$

where $c_n$ is a weight of the *n*th sample. This penalization form with $\lambda =\frac{1}{2C}$ is the same as

$$\begin{aligned}&\text {min}_{w,b}\frac{1}{2C} \Vert w \Vert ^2 + \sum _{n=1}^N c_n [1- y_n (\langle w , x_n \rangle + b)]_+ \nonumber \\&\quad =\text {min}_{w,b} \frac{1}{2} \Vert w \Vert ^2 + C \sum _{n=1}^N \xi _n, \end{aligned}$$

subject to the constraints $\xi _n \ge 0, \ \xi _n \ge c_n(1-y_n (\langle w , x_n \rangle + b))$. Consider the soft margin SVM. Let

4 $$\begin{aligned} Q(\beta ,\xi )=\frac{1}{2} \Vert w \Vert ^2 + C \sum _{n=1}^N \xi _n \end{aligned}$$

and

5 $$\begin{aligned} R(\beta )= \frac{1}{2} \Vert w \Vert ^2 + C \sum _{n=1}^N c_n [1- y_n h(x_n;\beta ) ]_+, \end{aligned}$$

where $\beta =(w,b)$ and $h(x;\beta )=\langle w , x \rangle +b$. Equivalence between (4) and (5) is proved in Lemmas 1 and 2.

#### **Lemma 1**

*Let*$\xi _n^* = c_n [1- y_n h(x_n;\beta ) ]_+$*for*$n=1,\dots ,N$*and*$c_n \ge 0$. *Then, I get*

$$\begin{aligned} \xi _n^* = \text {argmin}_\xi Q (\beta , \xi ), \end{aligned}$$

*subject to*$\xi _n^* \ge 0$ and $\xi _n^* \ge c_n [1- y_n h(x_n;\beta ) ]_+$*for*$n=1,\ldots ,N$. *The details of the proof are presented in Additional file*1.

**Table 1:** The weighted KM-SVM (or SVM) with the boosting algorithm

1. Initialize the weight $c_n$ with $\frac{1}{N}$.	
2. For $m=1$ to M:	
(1) Fit a KM-SVM (or SVM) $G_m(x)$ with weights $c_n$ to clustering centers of train data.	
(2) Compute	
$$err_m = \frac{\sum _{n=1}^N c_n I \big (y_n \ne G_m(x_n) \big ) }{\sum _{n=1}^N w_n }$$	
(3) Compute $\alpha _m = \text {log} \big ( \frac{1-err_m}{ err_m } \big )$	
(4) Set $c_n \leftarrow c_n \cdot \text {exp} \big [ \alpha _m \cdot I (y_n \ne G_m(x_n) ) \big ]$	
3. Output $G(x) = \text {Sign}\Big [ \sum _{m=1}^M \alpha _m G_m(x) \Big ]$.	

#### **Lemma 2**

*Let*$(\beta ^*, \xi ^*)$*be the minimizer of*$Q(\beta , \xi )$*subjectto* (3.5). *I obtain*

$$\begin{aligned} \beta ^* = \text {argmin}_\beta R(\beta ). \end{aligned}$$

*Hence*, (4) *is derived by optimizing* (5) *with respect to*$\xi$. *See the details of the proof in Additional file*1.

### Solutions for weighted SVM

In this section, I derive the solution of the weighted SVM. I adopt the quadratic programming (QP) to solve for some $C > 0$,

6 $$\begin{aligned} min_{w,b,\xi }\frac{1}{2} \Vert w \Vert ^2 + C \sum _{n=1}^N \xi _n, \end{aligned}$$

subject to the constraints $\xi _n \ge 0$ and $\xi _n \ge c_n \big ( 1 - y_n(\langle w , x_n \rangle + b) \big )$ for $n=1, \dots , N$. Consider the Lagrangian

7 $$\begin{aligned} L(w,b,\xi ,\alpha ,r) = \frac{1}{2} \Vert w \Vert ^2 + C \sum _{n=1}^N \xi _n - \sum _{n=1}^N \alpha \big \{ c_n y_n \big ( \langle w , x_n \rangle + b \big ) - c_n + \xi _n \big \} - \sum _{n=1}^N r_n \xi _n. \end{aligned}$$

With a little of algebra, I can build the Wolfe dual form to estimate the weight term *w* and *b*, and it is enough to solve the dual problem as below:

$$\begin{aligned} \text {Maximize} \ \sum _{n=1}^N \alpha _n \ c_n - \frac{1}{2} \sum _{n=1}^N \sum _{m=1}^N \alpha _n \ c_n y_n \alpha _m \ c_m y_m \langle x_m , x_n \rangle , \end{aligned}$$

subject to $\sum _{n=1}^N \alpha _n \ c_n y_n = 0$ and $0 \le \alpha _n \le C$ for $n=1,\dots ,N$. See the details of the proof in Additional file 1.

### Weighted KM-SVM with an ensemble technique

Generally it is known that the KM-SVM boosts computational efficiency at the expense of prediction accuracy. Such low accuracy of KM-SVM can be overcome with importing ensemble methods (e.g., boosting Schapire 1990; Breiman 1998), and these ensemble methods can be applicable to the standard SVM as well. In this paper, I make use of AdaBoost.M1 introduced by Freund and Schapire (1997). In principle, AdaBoost.M1 increases weights to mis-classified samples. At each boosting iteration, weighted weak classifiers are stacked by samples, and produces integrated classification rules by majority voting. Simply put, the weighted KM-SVM (and wSVM) is more of applying boosting to weights in order to add an artificial impact to mis-classified samples. The following is the weighted KM-SVM (and wSVM) objective function (3):

$$\begin{aligned} \text {min}_{w,b} \sum _{n=1}^N c_n [1- (y_n \langle w , x_n \rangle + b)]_+ + \lambda \Vert w \Vert ^2, \end{aligned}$$

The weight $c_n$ is updated via $c_n \cdot \text {exp} \big [ \alpha _m \cdot I (y_n \ne G_m(x_n) ) \big ],$ where $\alpha _m = \text {log} \big ( \frac{1-err_m}{ err_m } \big )$ and $err_m = \frac{\sum _{n=1}^N c_n I \big (y_n \ne G_m(x_n) \big ) }{\sum _{n=1}^N w_n }$. Table 1 summarizes the algorithm of the weighted KM-SVM (and SVM) with the boosting method. At each iteration $(1 \le m \le M)$, I fit a KM-SVM weak classifier $G_m(x)$ together with the weighted term $c_n$ as in Step 2-(1). The weighted error rate ($=err_m$) is then calculated in Step 2-(2). In Step 2-(3), I calculate the weight constant $\alpha _m$ given $G_m(x)$. It is worthwhile to note that weights of clustering centers misclassified by $G_m(x)$ increases by exp($\alpha _m$). In other words, $\alpha _m$ serves to adjust relative importance of misclassified samples. In Step 2-(4), I finalize the classifier *G*(*x*) by integrating all weak classifiers via majority voting.

## Numerical studies

### Simulated data

**Fig. 1:**
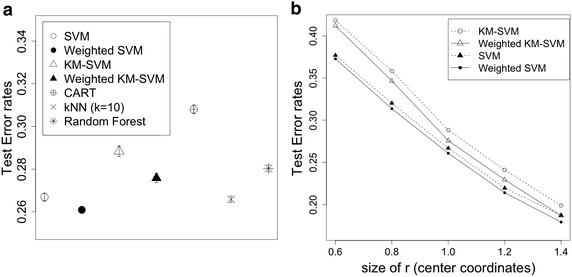
Performance comparisons across different classification rules. *Each dot* represents the averaged values of repeated simulations, and the *bars* overlaid with *dots* represent standard errors. **a** Prediction errors of six different classification rules, **b** decreasing patterns of test error rates as *r* (coordinates of centers) increases in value

**Fig. 2:**
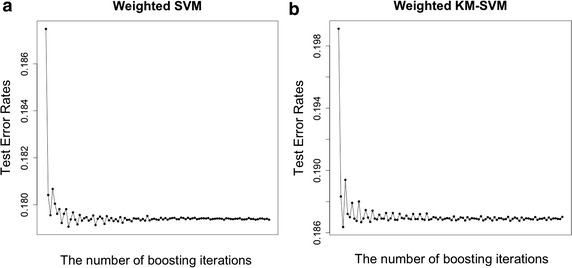
**a** Test error rates of the weighted SVM as the boosting increases in iteration. **b** Test error rates of the weighted KM-SVM as the boosting increases in iteration

In this section, I examine predictive performance of the weighted KM-SVM (and SVM) with boosting. Below I briefly illustrate how I generate simulated data. Let $y_n \in \{ -1, 1 \}$ be the binary variable of the $n^{th}$ sample ($y_n = -1 \ \text {for} \ 1\le n \le \frac{N}{2}$; $y_n = 1$ for $\frac{N}{2} + 1 \le n \le N$), and $X \in {\mathbb {R}}^{N \times 2}$ be a matrix of two predictor variables $(x_{n}^1, x_{n}^2)$ randomly generated from the bivariate normal distribution, where $\mu = (0,0) \ \text {for} \ 1 \le n \le \frac{N}{2}$ and $\mu = (r,r)$ for $\frac{N}{2} + 1 \le n \le N$, $r=2$ and $\Sigma = I$. With the simulation scheme above, I generated $N=100$ samples for train data and $N=1000$ samples for test data. The regularization parameter *C* was chosen by 5-fold cross-validation over $2^{-5},\ldots , 2^{5}$ from train data, and the radial based kernel was applied with $\sigma =1$ (a.k.a. free parameter). The number of clusters (*K*) is defined by half size of train data. Making use of the weighted KM-SVM (and SVM) fitted by the optimal parameter, I calculated error rates of test data. The experiment to generate test error rates (= error rates of test data) was repeated 1000 times and average values are presented in Fig. 1a, b. The test error rates were benchmarked to compare with other classification rules. In Fig. 1a, I first observe that the SVM (= 0.265) performs better than the KM-SVM (= 0.291) in accuracy. This is consistent with previous experimental knowledge (Lee et al. 2007). In addition, I notice that the weighted KM-SVM (= 0.278) (and SVM = 0.265) considerably improves the non-weighted KM-SVM (= 0.291) (and SVM = 0.26). Generally, the SVM is believed to be superior to many popular prediction rules. In this simulation, I consider CART (Breiman et al. 1984), kNN (Altman et al. 1992) and Random forest (Ho 1998) for comparison with the family of SVM classifiers. In Fig. 1a, the weighted SVM performs best among all of classification rules. Moreover, it is remarkable to see that the weighted KM-SVM performs better than CART and Random forest despite its data reduction. Figure 2a, b illustrate how the proposed methods reduce error rates as iterated. The test error rates dramatically drop after the first few iterations, and hence boosting evidently helps increasing accuracy. In Fig. 1b, the declining pattern of test error rates are presented as *r* (i.e., a parameter for $\mu$ for $\frac{N}{2} + 1 \le n \le N$) increases in size ranging from 0.6 to 1.4. It is clear to say that the weighted KM-SVM (and wSVM) is consistently better than the KM-SVM (and SVM).

**Table 2:** Shown are the brief descriptions of the nineteen microarray datasets of disease-related binary phenotypes (e.g., case and control). All datasets are publicly available

Name	Study	Type	# of samples	Control	Case	# of matched genes	Reference
BRCA	Breast cancer	Methylation	343	27	316	10,121	The Cancer Genome Atlas (TCGA)
KIRC	Kidney cancer	Methylation	418	199	219	10,121	The Cancer Genome Atlas (TCGA)

### Application to genomic data

**Fig. 3:**
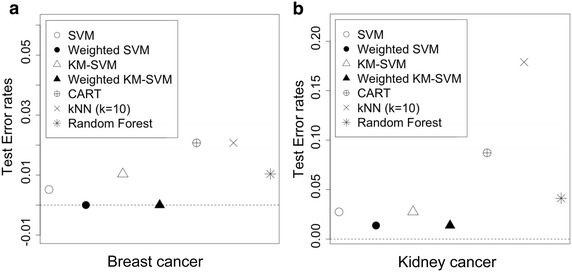
**a** Performance comparisons of breast cancer data across different classification rules, **b** performance comparisons of kidney cancer data across different classification rules

Below I demonstrate applications to two real methylation expression profiles for breast and kedney cancer. TCGA cancers data (Level 3 DNA methylation of beta values targeting on methylated and the unmethylated probes) from the TCGA database (https://tcga-data.nci.nih.gov/tcga/), where I retrieved methylation data of two cancer types (Breast carcinoma (BRCA), Kidney renal clear cell carcinoma (KIRC). I matched up features across all studies and filtered out probes by the rank sum of mean and standard deviation (Wang et al. 2012) (mean <0.7, SD <0.7), which leaves 910 probes. Table 2 describes details of TCGA data. In this application, I pose a hypothetical question if the proposed methods (wKM-SVM and wSVM) can improve accuracy for cancer prediction. To this end, I first randomly split the whole data set into two parts with approximately same size, which I denote as train and test data. The number of clusters (*K*) is defined by half size of train data. I examined by the test set weighted KM-SVM’s (and wSVM’s) performance using each SVM constructed by the train set. Similar to simulation studies, I observe that the weighted KM-SVM (and wSVM) outperforms the standard KM-SVM (and SVM) in prediction accuracy. It is also notable that the weighted KM-SVM (and wSVM) better performs than CART, kNN and Random Forest, as shown in Fig. 3a, b. Therefore, I conclude that the proposed weighted SVM can facilitate cancer prediction with enhanced accuracy.

## Conclusion and discussion

In this paper, I propose the new algorithm for the weighted KM-SVM to improve prediction accuracy. Typically, the KM-SVM has higher error rate than that it appears in the SVM, due to data reduction. To circumvent this issue, I suggest the weighted KM-SVM (and SVM) and evaluated performance of each of classifiers through various experimental scenarios. Putting together, I conclude that the proposed weighted KM-SVM (and SVM) is effective to diminish its error rates. In particular, I applied the weighted KM-SVM (and SVM) to TCGA cancer methylation data, and found its improved performance for disease prediction. Due to high accuracy, the weighted KM-SVM (and wSVM) can be widely used to facilitate predicting the complex diseases and therapeutic outcomes. Looking beyond this scope, this precise classification rule advances the upcoming horizon in pursuit of precision medicine, as it is urgently required in the bio-medical field to identify relations between bio-molecular units and clinical phenotype patterns (e.g., candidate biomarker detection, disease subtypes identification and associated biological pathways). The KM-SVM, however, does not involve size of clusters (i.e., the number of samples that belong to a cluster), and so clustering centers may not suitably represent original data structures. This weakness point may potentially results in poor prediction. For future work, I may suggest a new weighting scheme in proportion to size of clusters to improve more in accuracy. I leave this idea to next study.

## Additional file

10.1186/s40064-016-2677-4 Numerical verification for the weighted support vector machine.
