# Supplementary material for: Weighted K-means support vector machine for cancer prediction
Source: Springerplus. 2016 Jul 25;5(1):1162. doi: 10.1186/s40064-016-2677-4 (PMC4960100; doi:10.1186/s40064-016-2677-4)
Supplement: Supplementary file 1 — 10.1186/s40064-016-2677-4 Numerical verification for the weighted support vector machine. [file 40064_2016_2677_MOESM1_ESM.pdf]

Supplementary material: Weighted K-means Support Vector Machine for Cancer Prediction

SungHwan Kim[1]

[1] Department of Statistics, Korea University, Seoul, South Korea

December 21, 2015

1 Numerical verification for the weighted support vector machine

1.1 Proof for Lemma 1

ξ_n^* satisfies below conditions

$$\xi_n^* \geq 0 \text{ and } \xi_n^* \geq c_n[1 - y_n h(x_n; \beta)]_+ \text{ for } n = 1, \ldots, N.$$

Let's take any ξ satisfying (3.5) conditions. Then,

$$\xi_n^* \geq c_n[1 - y_n h(x_n; \beta)]_+ = \xi_n^*$$

Hence, Lemma 1 is proved as below:

$$\begin{aligned} Q(\beta, \xi^*) &= \frac{1}{2}\|w\|^2 + C\sum_{n=1}^{N} \xi_n^* \\ &\leq \frac{1}{2}\|w\|^2 + C\sum_{n=1}^{N} \xi_n \\ &= Q(\beta, \xi). \end{aligned}$$

1.2 Proof for Lemma 2

It is clear that

$$\beta^* = \text{argmin}_\beta Q(\beta, \xi^*) = \text{argmin}_\beta R(\beta).$$

1.3 Proof for the solution to the wSVM.

In order to solve non-separable case, one introduce slack variables,

$$\xi_n \geq 0 \text{ for } n = 1, \ldots, N \tag{1}$$

and use relaxed separability constraints with a weighted term c_n

$$\xi_n \geq c_n\big(1 - y_n(\langle w, x_n\rangle + b)\big) \tag{2}$$

$$\Leftrightarrow c_n y_n\big(\langle w, x_n\rangle + b\big) \geq c_n - \xi_n \text{ for } n = 1, \ldots, N. \tag{3}$$

SVM uses the quadratic programming (QP) to solve, for some $C > 0$,

$$min_{w,b,\xi} \frac{1}{2}\|w\|^2 + C\sum_{n=1}^{N}\xi_n, \tag{4}$$

subject to the constraints (1) and (2).

Consider the Lagrangian

$$L(w,b,\xi,\alpha,r) = \frac{1}{2}\|w\|^2 + C\sum_{n=1}^{N}\xi_n - \sum_{n=1}^{N}\alpha\big\{c_n y_n\big(\langle w, x_n\rangle + b\big) - c_n + \xi_n\big\} - \sum_{n=1}^{N} r_n\xi_n. \tag{5}$$

From

$$\begin{aligned}
\frac{\partial L}{\partial w} &= w - \sum_{n=1}^{N}\alpha_n c_n y_n x_n = 0\\
\frac{\partial L}{\partial \xi_n} &= C - \alpha - r_n = 0\\
\frac{\partial L}{\partial b} &= \sum_{n=1}^{N}\alpha_n c_n y_n = 0,
\end{aligned}$$

we have

$$w^* = \sum_{n=1}^{N}\alpha_n c_n y_n x_n. \tag{6}$$

Since $0 < r_n = C - \alpha_n$, we have $\alpha_n < C$. Note that

$$\begin{aligned}
\langle w^*, w^*\rangle &= \sum_{n=1}^{N}\sum_{m=1}^{N}\alpha_n c_n y_n \alpha_m c_m y_m \langle x_m, x_n\rangle\\
\langle w^*, x_n\rangle &= \sum_{n=1}^{N}\alpha_m c_m y_m \langle x_m, x_n\rangle.
\end{aligned}$$

Hence,

$$
\begin{aligned}
g(\alpha, r) &= L(w^*, b^*, \xi^*, \alpha, r) \\
&= \frac{1}{2}\|w^*\|^2 + C\sum_{n=1}^{N}\xi_n^* - \sum_{n=1}^{N}\alpha\big\{c_n y_n\big(\langle w^*, x_n\rangle + b^*\big) - c_n + \xi_n^*\big\} - \sum_{n=1}^{N} r_n\xi_n^* \\
&= \frac{1}{2}\|w^*\|^2 - \sum_{n=1}^{N}\alpha_n\big\{c_n y_n\big(\langle w^*, x_n\rangle + b^*\big) - c_n\big\} + \sum_{n=1}^{N}(C - \alpha_n - r_n)\xi_n^* \\
&= \frac{1}{2}\|w^*\|^2 - \sum_{n=1}^{N}\alpha_n\big\{c_n y_n\big(\langle w^*, x_n\rangle + b^*\big) - c_n\big\} \\
&= \frac{1}{2}\|w^*\|^2 - \sum_{n=1}^{N}\alpha_n\ c_n y_n\langle w^*, x_n\rangle - b^*\sum_{n=1}^{N}\alpha_n\ c_n y_n + \sum_{n=1}^{N}\alpha_n c_n \\
&= \frac{1}{2}\sum_{n=1}^{N}\sum_{m=1}^{N}\alpha_n\ c_n y_n \alpha_m\ c_m y_m\langle x_m, x_n\rangle \\
&- \sum_{n=1}^{N}\alpha_n\ c_n y_n\Big(\sum_{m=1}^{N}\alpha_m\ c_m y_m\langle x_m, x_n\rangle\Big) - b^*\sum_{n=1}^{N}\alpha_n\ c_n y_n + \sum_{n=1}^{N}\alpha_n c_n \\
&= -\frac{1}{2}\sum_{n=1}^{N}\sum_{m=1}^{N}\alpha_n\ c_n y_n \alpha_m\ c_m y_m\langle x_m, x_n\rangle - b^* \times 0 + \sum_{n=1}^{N}\alpha_n\ c_n \\
&= W(\alpha).
\end{aligned}
$$
